# Effect of nanostructured lipid carriers on transdermal delivery of tenoxicam in irradiated rats

**DOI:** 10.1080/10717544.2020.1803448

**Published:** 2020-08-10

**Authors:** Saud Bawazeer, Dalia Farag A. El-Telbany, Majid Mohammad Al-Sawahli, Gamal Zayed, Ahmed Abdallah A. Keed, Abdelaziz E. Abdelaziz, Doaa H. Abdel-Naby

**Affiliations:** a Pharmaceutical Chemistry Department, Faculty of Pharmacy, Umm Al-Qura University, Makkah, Saudi Arabia; b Department of Pharmaceutics, Faculty of Pharmacy, Modern University for Technology and Information, Cairo, Egypt; c Department of Pharmaceutical Technology, Faculty of Pharmacy, Kafrelsheikh University, Kafrelsheikh, Egypt; d Department of Pharmaceutics and Industrial Pharmacy, Faculty of Pharmacy, Al-Azhar University, Assiut, Egypt; e Quality Control Department, AUG Pharma Company, 6 October City, Egypt; f Department of Drug Radiation Research, National Centre for Radiation Research and Technology, Atomic Energy Authority, Cairo, Egypt

**Keywords:** Tenoxicam, nanostructured lipid carriers, transdermal delivery, carrageenan, irradiated rats

## Abstract

Transdermal delivery of non-steroidal anti-inflammatory drugs (NSAIDs) is an effective route of drug administration, as it directs the drug to the inflamed site with reduced incidence of systemic adverse effects such as gastric hemorrhage and ulcers. Tenoxicam (TNX) is a member of NSAIDs that are marketed only as oral tablets due to very poor absorption through the skin. The current study intended to formulate and characterize a hydrogel loaded with nanostructured lipid carriers (NLCs) to enhance the transdermal delivery of TNX. Six formulations of TNX were formulated by slight modifications of high shear homogenization and ultrasonication method. The selected formula was characterized for their particle size, polydispersity index (PDI), zeta potential, entrapment efficiency (EE), *in-vitro* drug release and *ex-vivo* skin permeation studies. Moreover, the effectiveness of the developed formula was studied *in-vivo* using carrageenan-induced paw edema and hyperalgesia model in irradiated rats. Formula F4 was chosen from six formulations, as the average diameter was 679.4 ± 51.3 nm, PDI value of about 0.02, zeta potential of −4.24 mV, EE of 92.36%, globules nanoparticles without aggregations and absence of interactions in the developed formula. Additionally, the *in-vivo* study showed the efficacy of formula F4 (TNX-NLCs hydrogel) equivalent to oral TNX in reducing the exaggerated inflammatory response induced by carrageenan after irradiation. In conclusion, the present findings suggest that TNX-NLCs hydrogel could be a potential transdermal drug delivery system alternative to the oral formulation for the treatment of various inflammatory conditions.

## 1. Introduction

Tenoxicam (TNX) is one of the potent non-steroidal anti-inflammatory drugs (NSAIDs) of the oxicam class, used to treat inflammation and pain associated with rheumatoid arthritis and other joint diseases (Bird, 1987). Similar to most NSAIDs, oral intake of TNX is associated with gastrointestinal adverse effects such as heartburn, nausea, diarrhea, and vomiting (Gonzalez & Todd, 1987). These adverse effects supported the search for another route of drug administration to avoid or minimize such unwanted effects.

The drug delivery through the skin was proved to be a convenient and effective route of drug administration since most inflammatory diseases occur locally near the body surface. Transdermal application of NSAIDs on the inflamed site can, therefore, offer the benefit of directing the drug to the inflamed site, producing its systemic effect, and obviating the adverse effects related to oral administration (Cevc & Blume, 2001; Baranowski et al., 2018). However, studies on topical application of TNX have shown that its penetration is very poor; as a result, the drug is mostly available as conventional oral formulations (Karadzovska et al., 2013; Negi et al., 2014).

Nanostructured lipid carriers (NLCs) and solid lipid nanoparticles were developed as drug delivery carriers through the skin because they are lipid in nature, biocompatible and composed form nontoxic and irritant materials. The NLCs are expected to release the loaded drug directly into the systemic circulation with minimum side effects. Additionally, NLCs are characterized by large surface area which enables longer contact time of the drug with the skin for sustained drug delivery (Sharma et al., 2013; Kurakula et al., 2016). NLCs are relatively cheap, biocompatible, and suitable for the incorporation of lipophilic as well as hydrophilic drugs and they also can improve the stability of the incorporated drug (Kurakula et al., 2016). Moreover, using hydrogel as a delivery system can increase the residence time of drugs on the skin surface and provide a faster release of the loaded drug (Karadzovska et al., 2013; Abdellatif et al., 2019).

Various experimental models of inflammation, such as carrageenan-induced paw edema (Khayyal et al., 2009; El-Ghazaly et al., 2017) and adjuvant-induced arthritis (El-Ghazaly & Khayyal, 1995; El-Ghazaly et al., 2011), reported that ionizing radiation increased the release of inflammatory mediators. For screening anti-inflammatory drugs, carrageenan-induced paw edema has been widely used as an acute animal model of inflammation.

From this point of view, the present study aimed to enhance the transport of TNX through the skin by loading into NLCs hydrogel and to evaluate its effectiveness using *in-vitro* and *ex-vivo* skin permeation assessments. Our aim was also extended to estimate the *in-vivo* efficacy of TNX-NLCs hydrogel in reducing the radiation-induced exaggeration of the inflammatory response induced by carrageenan and to present it as a possible replacement for the oral NSAID formulations.

## 2. Materials and methods

### 2.1. Materials

TNX was kindly supplied from Egyptian International Pharmaceutical Company (EIPICO), Tenth of Ramadan City, Egypt. Compritol 888 ATO was kindly supplied from Gattefosse, France. Isopropyl myristate (IPM) was obtained from Merck Schuchardt (Hohenbrunn, Germany). All other chemicals and reagents were purchased from Sigma-Aldrich (Saint Louis, MO) such as Pluronic F68, Pluronic F127 and Carrageenan.

### 2.2. Formulation of TNX-NLCs

Six NLCs formulations (F1-F6) containing TNX were prepared by slight modifications of high shear homogenization and ultrasonication method (Ghasemiyeh & Mohammadi-Samani, 2018; Mishra et al., 2018; Sarangi et al., 2019). The lipid phase composed of Compritol 888 ATO as solid lipid and Isopropyl myristate as liquid lipid. Formulations compositions were illustrated in Table 1. The lipid phase was heated to 5 °C above the melting point of the used lipid. To obtain a drug-lipid mixture, the drug was dissolved in the heated lipid phase. The aqueous phases consisted of Pluronic F68 or Pluronic F127 were warmed to almost the same temperature of the lipid phase. The hot lipid phase was then poured onto the hot aqueous phase and homogenized with a high-speed homogenizer (Heidolph Homogenizer, Heidolph Instruments, Germany) at 12,000 rpm for 10 min. The resulted hot o/w emulsion was sonicated in a water bath for 30 min. and then cooled to room temperature. Drug-free NLCs (control) were prepared by the same procedure.

**Table 1.** Composition of prepared nanostructured lipid carriers containing tenoxicam.

Formula	Solid lipid	Liquid lipid	Solid lipid:liquid lipid	Hydrophilic emulsifier
F1	Compritol 888 ATO	Isopropyl myristate	8:2	Pluronic F68
F2			7:3	
F3			6:4	
F4			8:2	Pluronic F127
F5			7:3	
F6			6:4	

### 2.3. Characterization of TNX-NLCs

Dynamic light scattering (DLS) was utilized to measure the particle size, polydispersity index (PDI), and zeta potential using particle size analyzer (Malvern Series ZS90, Malvern Co., UK) at 25 °C. In addition, entrapment efficiency (EE%) was estimated by measuring the concentration of unloaded TNX in the dispersion medium. The unentrapped TNX was determined by adding 0.5 ml of TNX-NLCs to 9.5 ml Sorenson buffer. This dispersion was cold centrifuged at 14,000 rpm for 1 h. Afterward, the supernatant was filtered through 0.22 μm Millipore membrane filter and analyzed for un-encapsulated TNX at 368 nm using UV-Vis spectrophotometric method (SHIMADZU-1700 UV, Japan, UVPC personal spectroscopy, software version 2.32) after suitable dilution (Hou et al., 2003). EE% was calculated according to the following equation:

(1) $$\text{EE}\%=\frac{\text{Total}\;\text{drug}\;\text{amount}\;–\;\text{Unentrapped}\;\text{drug}\;\text{amount}}{\text{Total}\;\text{drug}\;\text{amount}}\;\times \;100$$

### 2.4. *In-vitro* drug release study

The *in-vitro* release of TNX was evaluated by the Sample and Separate method (Shen & Burgess, 2013; D’Souza, 2014). Nanoparticulate dosage form was introduced into the release media that was maintained at a constant temperature (±37 °C) using the type II dissolution apparatus (Erweka^®^, Heusenstamm, Germany). The dissolution medium was 250 ml of Sorenson phosphate buffer (pH 7.4) and stirred at 50 rpm. Samples (0.3 ml) were withdrawn at the time intervals 15, 30, 60, 90, 120, 150, 180 min, and then replaced with fresh medium to keep the volume of release medium constant (Heng et al., 2008). Each sample was poured into a disposable microcuvette and analyzed for drug content at the *λ*_max_ (368 nm) by a UV-Vis spectrophotometer. Each experiment was carried out three times and according to release study outcomes, one formula for further investigation was selected.

### 2.5. Transmission electron microscopy (TEM)

TEM (JEM-2100; JEOL, Japan) operating at 100 kV was used to assay the morphological examination of the prepared NLCs. One drop from the diluted formula was taken and placed over a copper grid coated with carbon. It was then negatively stained by adding one drop of an aqueous solution with phosphotungstic acid (1% w/v). Filter paper wiped off the excess staining solution and allowed drying at room temperature (Hashem et al., 2015).

### 2.6. Fourier transform infrared spectroscopy (FTIR)

The FTIR spectra of TNX-NLCs were recorded using FTIR spectroscopy (Bruker, model alpha, Germany). Samples were mixed with potassium bromide (spectroscopic grade) and compressed into disks using hydraulic press before scanning from 4000 to 600 cm^−1^ was carried out according to El-Feky et al. (2020).

### 2.7. Preparation and evaluation of TNX-NLCs hydrogel

Hydrogels composed of 5% w/w sodium carboxymethylcellulose (Na-CMC) and hydroxypropyl methylcellulose (HPMC) were prepared by dispersing the hydrogelling agents in water under stirring. The obtained hydrogel was added to F4 formula dispersion and pure TNX and mixed at 800 rpm (Magnetic stirrer, Velp Scientifica, Italy). Stirring was continued till dispersion and hydrogel containing 1% w/w of TNX was formed. TNX-NLCs hydrogel was stored at 4 °C until use. The appearance and other physical properties, including clarity, turbidity, and precipitation of freshly prepared hydrogels were inspected (Qi et al., 2007). *In-vitro* TNX release from NLCs based hydrogel was evaluated as described before. Syringe filters with a pore size as large as 0.22 μm have been used for withdrawing supernatant to monitor drug release (Heng et al., 2008).

### 2.8. Animals and ethical considerations

Animal care and handling were done in conformity with the ethical guidelines organized by the European Communities Council Directive (86/609/EEC). Male Westar rats weighing (130–150 g) were got from the animal breeding unit of the National Center for Radiation Research and Technology (NCRRT)-Atomic Energy Authority (Nasr City, Cairo, Egypt). They were housed under managed environmental conditions throughout the experimental period, allowed standard laboratory chow and water ad libitum.

### 2.9. *Ex-vivo* skin permeation study

The rats’ abdominal hair was shaved, then they were sacrificed under light ether anesthesia, and afterward, the abdominal skin was surgically excised. The dermal surface of the skin was cleaned by isopropyl alcohol to remove sub-cutaneous fats without damaging the epidermal surface. Subsequently, the skin was washed with distilled water then stored at 2 °C and used within one day. The *ex-vivo* experiment was carried out as described by AbdelSamie et al. (2016) using an experimental cell similar to the Franz diffusion cell. The prepared skin was first hydrated with phosphate buffer and slightly stretched over the end of the glass cylinder with a cross-sectional area of 7.07 cm^2^ (Yamaguchi et al., 1997). A specified amount of F4 formula using HPMC as a gelling agent, containing 20 mg of the drug was placed on the donor compartment of the diffusion cell subsequently immersed in a glass beaker containing 250 ml of phosphate buffer (pH 7.4) kept in a thermostatically controlled water bath at ± 37 °C and stirred at 50 rpm. At a certain time interval of up to 3 h, 4 ml of the diffusion medium was withdrawn and analyzed for TNX content by measuring the absorbance at *λ*_max_ 368 nm. To keep the vessel’s volume constant, an equal volume of fresh buffer was added to the diffusion medium and each experiment was repeated three times.

The TNX flux (*J*) across the rat skin was calculated by dividing the slope of the straight line of the amount against time by the permeation surface area. Additionally, the permeation coefficient (*K_p_*) of the drug was obtained applying Fick’s first law of diffusion according to the following equation:

(2) $$K_{p}=J/C$$

Where; *J* is the flux (mg/cm^2^/h) and *C* is the total TNX concentration in donor partition.

### 2.10. *In-vivo* study

#### 2.10.1. Carrageenan-induced paw edema model

A volume of 0.1 ml carrageenan suspension (1% w/v in saline) was injected into the sub-plantar surface of the rat right hind paw in rats using the method described in Winter et al. (1962). At the lateral malleolus level, the paws were marked with ink to ensure constant paw volume. The volume of the paw was then determined at 0 (*V_i_*) and 3 h after carrageenan injection (*V_f_*) using the Plethysmometer 7500 (Panlab Harvard, Barcelona, Spain). The paw volume increase was determined by the formula as a percentage of edema compared to the initial paw volume:

(3) $$\%\;\text{of}\;\text{Edema}\;=\;\left[\left(V_{f}-\;V_{i}\right)/V_{i}\right]\;\times \;100$$

While the inflammation severity inhibition percentage was calculated using the following formula:

(4) $$\%\;\text{of}\;\text{inhibition}\;=\;\left[\left(1\;–\;V_{t}/V_{c}\right)\right]\;\times \;100$$

Where, *V_t_* and *V_c_* represent mean edema volume of the carrageenan injected paw of drug-treated and control groups, respectively.

#### 2.10.2. Carrageenan-induced inflammatory paw hyperalgesia

The model of acute inflammatory pain was conducted according to the method described by Randall & Selitto (1957). This study was conducted similarly to the anti-inflammatory study, except that instead of edematous thickness, the pain threshold response of the right hind paw rat was measured. The withdrawal response latency time was estimated before carrageenan injection, and then using analgesic meters (Ugo Basile, Comerio, Varese, Italy) at 3 h. In response to carrageenan, the shortening of latency time was taken as an index of hyperalgesia. With a hind paw placed on a small plinth under a cone-shaped pusher with a rounded tip, each rat was gently held. The force applied in grams to the paw was steadily increased until the rat withdrew its paw. The pressure was removed immediately and the force needed to provoke the end-point response was recorded. A cutoff of 400 g was set to prevent mechanically induced injury.

#### 2.10.3. Irradiation of animals

Using the Gammacell^®^-40 biological irradiator with a source of Cesium-137 (Atomic Energy of Canada Limited; Sheridan Science and Technology Park, Mississauga, Ontario, Canada), the entire body irradiation of rats was carried out at NCRRT. In the plastic sample tray, non-anesthetized rats were placed and irradiated at 6 Gray (Gy) as a single exposure delivered at 0.41 Gy/min. The maximum time of animal exposure was about 14:30 min to achieve a radiation dose level of 6 Gy.

#### 2.10.4. Experimental design

Rats were randomly divided into seven groups, each of 6 rats as follow:

Group 1 (Normal): Saline was injected into the sub-plantar surface of rats’ right hind paw.
Group 2 (Inflamed): 0.1 ml of 1% carrageenan suspension was injected into the sub-plantar surface of rats’ right hind paw.
Group 3 (Inflamed irradiated): Rats were irradiated at a dose of 6 Gy and then inoculated with carrageenan after 24 h. (Şimşek et al., 2012).
Group 4 (Blank hydrogel): One gram of hydrogel was applied topically to the right hind paw of inflamed irradiated rats. The area of application was occluded with bandages and left for 3 h, after which the remaining hydrogel was wiped off the hind paws (Pawar & Pande, 2015).
Group 5 (Pure TNX hydrogel): 1% TNX hydrogel was applied to the inflamed irradiated rats by the same mode of application mentioned above.
Group 6 (TNX-NLCs hydrogel, F4): 1% TNX-NLCs hydrogel was applied to the inflamed irradiated rats using the same mode of application mentioned in group 4.
Group 7 (Oral TNX): TNX was administered as a single oral dose of 20 mg/kg to irradiated rats and after 1 h, carrageenan was injected into the rat right hind paw (Suleyman et al., 2008).

Three hours after induction by carrageenan, the paw volume, and nociceptive threshold were evaluated concurrently for the same experimental groups (Fernández-Dueñas et al., 2008). After that, the rats were sacrificed under light ether anesthesia by decapitation and each rat collected blood samples for reduced glutathione (GSH) determination. Serum was then separated from the blood and stored at −20 °C for the later measurement of thiobarbituric acid reactive substances (TBARS), total nitrate/nitrite (NOx), and tumor necrosis factor-alpha (TNF-α) later. Furthermore, biopsies of paws were removed and preserved in 10% formalin solution for histological examination.

#### 2.10.5. Histological examination

For histological examination, paw biopsies were embedded in paraffin wax, cut into 5 μm thick sections, and then stained with hematoxylin and eosin (H&E). The samples were examined under a Leica Aristoplan microscope (Leica, Bensheim, Germany) at 100× magnification, and images were taken with a charge-coupled device camera (Visitron Systems, Puchheim, Germany).

#### 2.10.6. Biochemical analysis

For the evaluation of oxidative stress biomarkers, reduced GSH was estimated in blood according to the method of Beutler et al. (1963). The method depends on the fact that both protein and non-protein SH-groups (mainly GSH) reacts with Ellman’s reagent [5,5′-dithiobis-(2-nitrobenzoic acid) (DTNB)] to form 5-thio-2-nitrobenzoic acid, which has a yellow color and can be measured colorimetrically at 412 nm using a Unicam 8625 UV/Vis spectrophotometer (Cambridge, UK). In addition, Uchiyama & Mihara (1978) method was used for the determination of lipid peroxidation in the serum. The method principle based on the colorimetric determination of a pink product resulting from the high-temperature reaction of TBARS with thiobarbituric acid in acid medium. The resulting color product is extracted using n-butanol and measured at 535 nm wavelength to calculate the content of TBARS as an index for lipid peroxidation.

NOx, an indicator of nitric oxide synthesis, was measured in serum according to Miranda et al. (2001). The assay depends on adding Griess reagent to convert nitrite into an azo compound with a bright reddish-purple color that can be measured at 540 nm colorimetrically.

As per the manufacturer’s instructions (ID Labs Biotechnology, London, Ontario, Canada), TNF-α, one of the pro-inflammatory cytokines, was measured in serum using a TNF-α ELISA kit specific to rats.

### 2.11. Statistical analysis

The *in-vitro* study data were expressed as mean ± standard deviation (SD). All other data, *in-vivo* study, were expressed as means ± standard error of the mean (s.e.m), and comparison between groups was conducted by one-way ANOVA followed by Tukey–Kramer multiple comparison test. The Kolmogorov-Smirnov (KS, *p* > .10) test proved data normal and the normal distribution of all data was found. Differences in mean values of less than 0.05 probability values (*p* < .05) were considered statistically significant. All statistical analysis was carried out using the software GraphPad Prism Version 7.0 (San Diego, CA).

## 3. Results

### 3.1. Formulation of TNX-NLCs

Liquid and solid lipids were selected for the preparation of NLCs based on their ability to carry the drug. A good affinity of solid and liquid lipids can warrant high EE%, which is an essential carrier system qualification (Negi et al., 2014). Compritol 888 ATO, with biodegradability properties and low toxicity, is a blend of different esters of behenic acid with glycerol. It has a melting point of 70.4 °C (Aburahma & Badr-Eldin, 2014). In a physically stable colloidal system, the solid lipid matrix plays a major role. IPM was used as a liquid matrix due to its good absorption through the skin. These lipid-based carrier systems were stabilized by Pluronic F68 or Pluronic F127 as a surfactant. TNX was incorporated at a constant weight of 0.01 g.

### 3.2. Characterization of TNX-NLCs

As shown in Table 2, the particle size was determined by DLS, ranged from 679.4 to 932.9 nm. This indicated that the method of NLCs formulation was successful to produce nanosized lipid carriers of TNX. PDI is a measure of colloidal system size distribution and is also considered an index that could indicate nanoparticles dispersion stability. The values of PDI recorded a range from 0.02 to 1. Ideally, the PDI value should be less than 0.70 as this value indicates a narrow particle size distribution that is, a monodisperse colloidal system. The zeta potential of the formulations was −4.24 to −7.59 mV.

**Table 2.** Characterization of prepared tenoxicam nanostructured lipid carriers’ formulations.

Formula	Particle size (nm)	Polydispersity index (PDI)	Zeta potential (mV)	Entrapment efficiency (EE %)
F1	775.3 ± 53.4	1	−6.32	96.64 ± 2.87
F2	896.8 ± 72.1	1	−6.57	94.14 ± 3.51
F3	932.9 ± 83.3	0.04	−7.59	92.22 ± 4.88
F4	679.4 ± 51.3	0.02	−4.24	92.36 ± 3.02
F5	867.6 ± 56.6	1	−4.84	92.61 ± 2.27
F6	930.6 ± 85.1	1	−5.23	92.69 ± 3.76

The particle size and entrapment efficiency (EE%) values were expressed as the mean ± SD.

Additionally, Table 2 shown that EE% of NLCs formulations were varied from 92.22 to 96.64%. The liquid lipid present in NLCs affects their EE% to a great extent by creating imperfections in a highly ordered crystal and consequently providing sufficient space for a large amount of drug to be loaded successfully (Salerno et al., 2010; Abdellatif et al., 2019).

### 3.3. *In-vitro* drug release study

**Figure 1.**
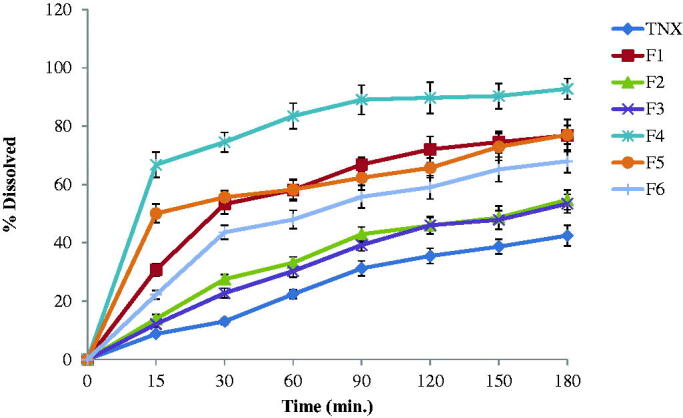
*In-vitro* release of tenoxicam (TNX) from tested formulations.

The *in-vitro* release results of TNX from NLCs tested formulations and pure TNX powder were shown in Figure 1 and represented as percent dissolved against time. The release of TNX form pure powder was lower than that from formulations containing drug in the form of NLCs. The release of TNX from the NLCs was studied. F4 formula exhibited the highest release profile. It exhibited 66.8% drug released in the first 15 min in comparison with 22.19% and 50.07% for pure drug and F5, respectively. F4 release recorded 74.53, 83.47, 89.7 and 92.81% after 30, 60, 120 and 180 min, respectively.

The released amounts of TNX were calculated from F4 showed a significantly higher TNX release (*p* < .05) from all other formulations.

Model-independent release parameters including release efficiency (RE), relative release rate (RRR), mean release time (MRT), percent released after 2 h (PR_120_), similarity factor *(f2*), and difference factor (*f1*) were calculated and listed in Tables 3 and 4. F4 formula exhibited the highest values of model-independent parameters while pure drug showed the lowest values of the same calculated parameters as shown in Table 3. Depending on *f2* (13, which is less than 50) and *f1* (201, which is higher than 15), the formulation of TNX in nanostructured lipid carriers greatly enhanced the *in vitro* dissolution of the drug (Zayed, 2014). Collectively, the F4 formula exhibited the smallest particle size (679.4 nm) and best release profile among the tested formulations. Based on the above results, the F4 formula was selected for further investigations on this study. The drug release kinetic analysis data which are shown in Table 4 is evidence that the release of TNX from the formulations of NLCs obeys the model of the first-order release.

**Table 3.** Model-independent parameters of tenoxicam (TNX) release from pure drug and different nanoparticle formulations.

Formula	Release parameters					
	RE%	RRR_120_	PR_150_	MRT	*f*2	*f*1
Pure TNX	27.21	1	38.7	24.82		
F1	60.89	2.03	74.53	48.77	23	122
F2	37.6	1.29	48.63	32.48	49	37
F3	35.55	1.3	47.88	31.19	54	29
F4	81.37	2.53	90.31	71.4	13	201
F5	60.73	1.85	72.9	55.67	23	126
F6	50.96	1.66	65.16	41.98	31	85
TNX-HPMC	42.4	1	53.13	33.44		
F4-HPMC	74.97	1.63	87.81	61.57	23	81
TNX-NaCMC	37.58	1	50.1	31.24		
F4-NaCMC	50.88	1.34	63.75	41.41	42	37

RE: release efficiency; RRR_120_: relative release rate after 2 h; PR_150_: percent released after 150 minutes; MRT: mean release time; *f*2: similarity factor; *f*: difference factor.

**Table 4.** Kinetic analysis of release data from tenoxicam (TNX) nanostructure lipid carriers.

Formula	*r*^2^			Mechanism	Intercept	Slope
	Zero order	First order	Diffusion			
TNX	0.9547	Diffusion	5.7502	3.6773	5.7502	3.6773
F1	0.8116		22.4643	4.3540	22.4643	4.3540
F2	0.9081		2.5672	3.9370	2.5672	3.9370
F3	0.9417		2.0244	4.2083	2.0244	4.2083
F4	0.8140		60.1074	2.6253	60.1074	2.6253
F5	0.9822		39.1614	2.6540	39.1614	2.6540
F6	0.8451		13.2577	4.2550	13.2577	4.2550

### 3.4. Transmission electron microscopy (TEM)

**Figure 2.**
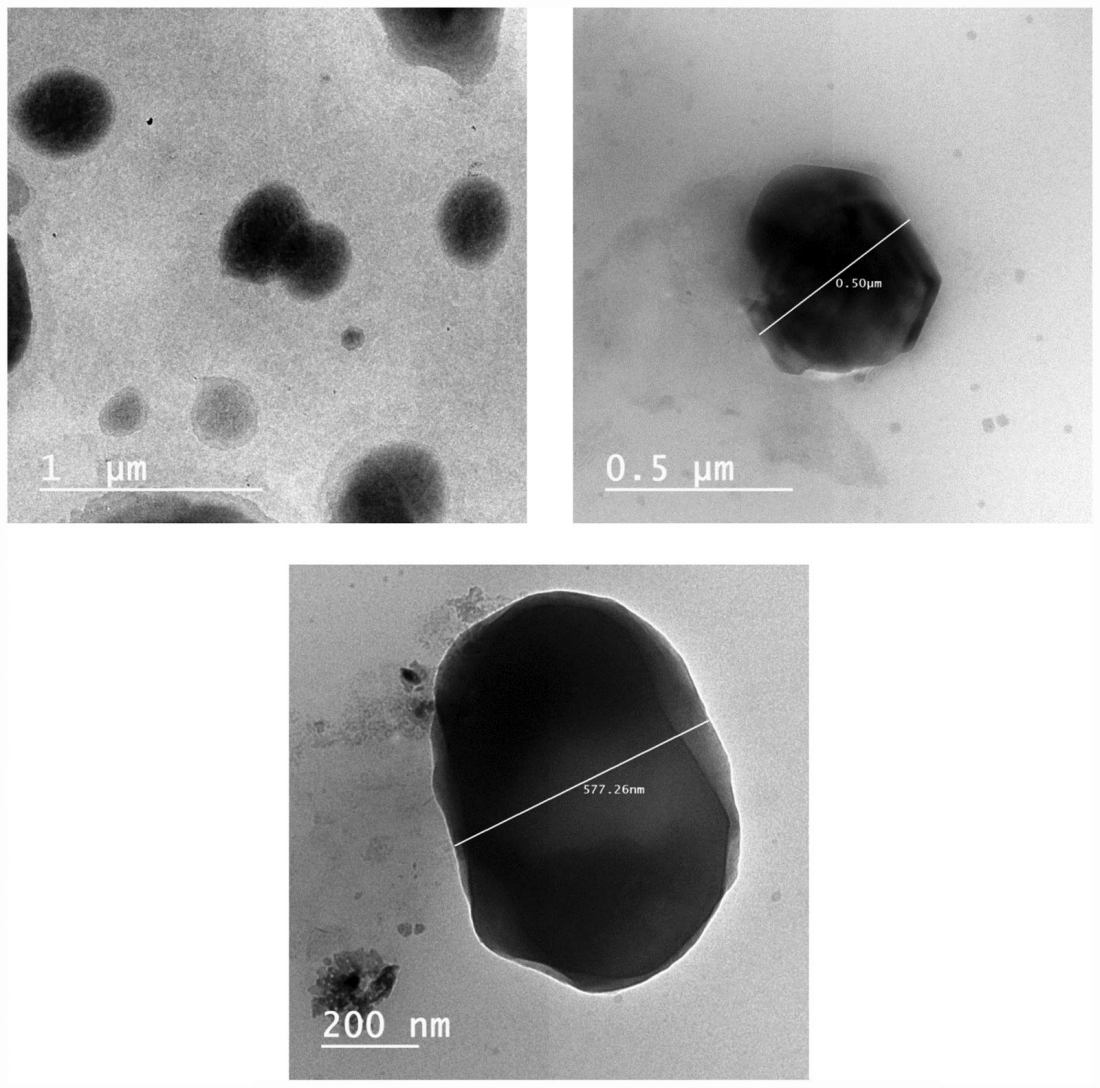
Transmission electron micrographs of the tenoxicam-nanostructured lipid carriers (TNX-NLCs, F4).

The TEM micrographs of TNX-NLCs selected F4 formula revealed that the nanoparticles are relatively almost globules, appeared as well dispersed black dots without any particle aggregation (Figure 2). The nanostructured size was consistent and their particles were well scattered without almost any aggregations. The particle sizes observed by TEM are smaller than the sizes determined by DLS because TEM determines the dense particle size while DLS measures the hydrodynamic sizes of the moving particles (particles and surrounding adsorbed layer of solvent and polymers) (Zayed & El-Feky, 2019).

### 3.5. Fourier transform infrared spectroscopy (FTIR)

**Figure 3.**
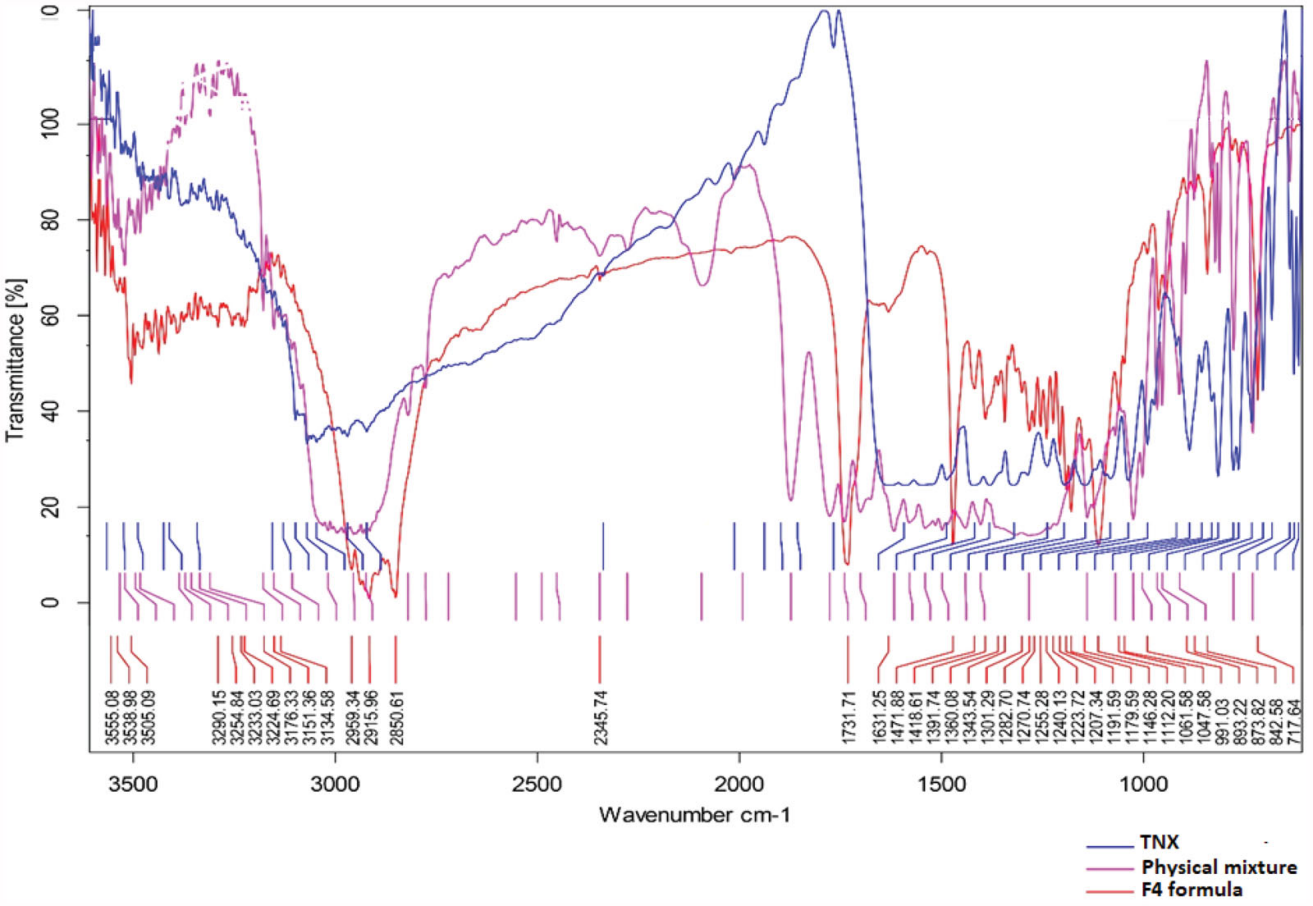
Fourier transform infrared spectroscopy (FTIR) spectra of pure tenoxicam (TNX), physical mixture of solid components, and F4.

Figure 3 showed the FTIR characteristics of TNX which these peaks not changed in the selected NLCs formula indicate that there is no interaction between drug and the used solid lipid.

### 3.6. Preparation and evaluation of TNX-NLCs hydrogel

**Figure 4.**
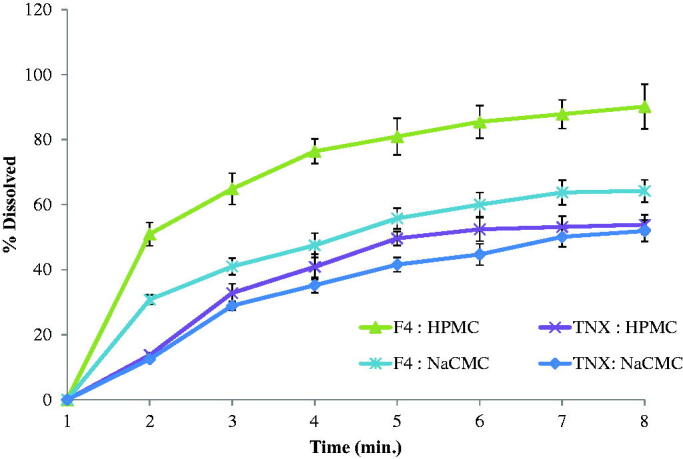
*In-vitro* drug release of tenoxicam (TNX) form the hydrogel.

TNX-NLCs and pure TNX were dispersed in two hydrogelling agents to evaluate the release behavior of the drug. Figure 4 described the pattern of TNX release from the suggested systems of the hydrogel. It was clear that the highest release profile observed with the HPMC hydrogel system loaded with TNX-NLCs. After 3 h, TNX-NLCs HPMC hydrogel showed a faster release profile than Na-CMC hydrogel due to the low viscosity of HPMC hydrogel.

### 3.7. Ex-vivo skin permeation study

**Figure 5.**
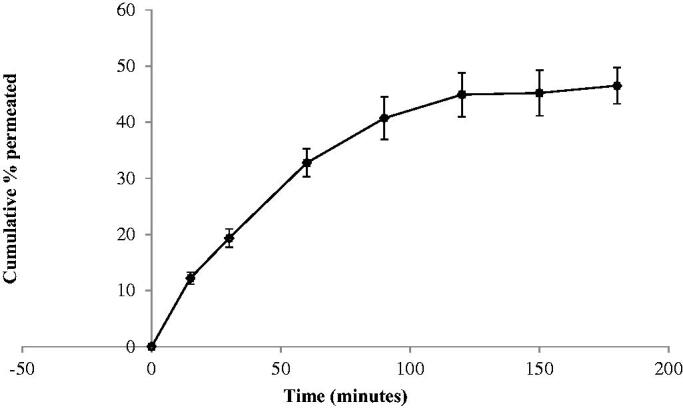
Skin permeation of tenoxicam (TNX) across natural rat skin using F4 formula.

The skin permeation profile of TNX across natural rat skin is presented in Figure 5. A higher amount of drug (about 50% of the loaded dose) was permeated across rat skin after 3 h. According to model-independent calculations, the release efficiency was higher form the gel base containing F4 nanoparticle than those containing the pure drug. The highest release efficiency (74.97) was observed for the HPMC gel base loaded with F4 nanoparticles. The tenoxicam flux, which indicates the *in-vivo* performance of the prepared formulation, was calculated at the end of permeation time and it was found equal to 12.37 × 10^−3 ^µg/cm^2^.

### 3.8. Effect of TNX-NLCs hydrogel against carrageenan-induced paw edema in irradiated rats

**Figure 6.**
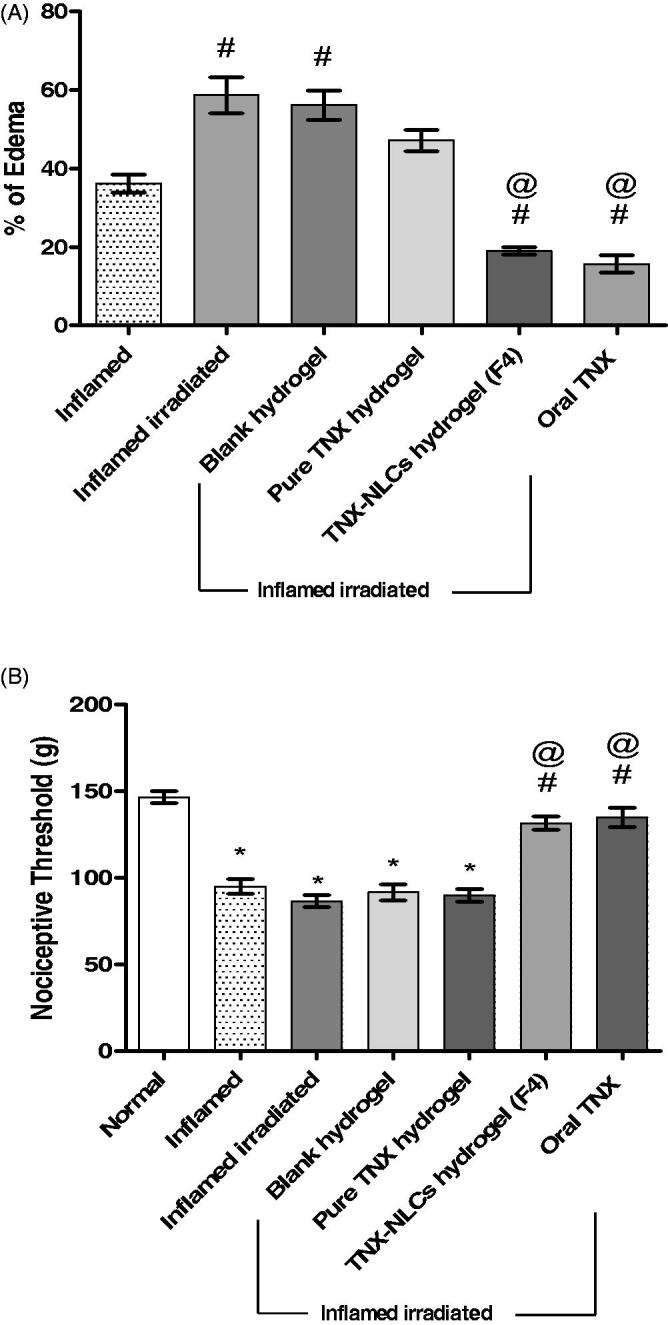
Effect of tenoxicam-nanostructured lipid carriers (TNX-NLCs) hydrogel against carrageenan-induced (A) paw edema and (B) hyperalgesia in irradiated rats. Statistical analysis was carried out by one-way ANOVA test. All values were expressed as mean ± s.e.m (*n* = 6). *Denotes statistical significance at *p* < .05 vs normal group. #denotes statistical significance at *p* < .05 vs inflamed group. @denotes statistical significance at *p* < .05 vs inflamed irradiated group.

The data presented in Figure 6(A), showed that the induction of carrageenan into the rat right hind paw resulted in a 36% increase in paw volume. Whereas, the paw volume markedly increased nearly by 59% after irradiation and carrageenan inoculation. The statistical analysis of this finding showed that the inflammatory response of carrageenan was significantly higher in irradiated rats compared to non-irradiated rats. Consequently, the inflamed irradiated rats were selected to study the anti-inflammatory effects of TNX. The application of the blank hydrogel and pure TNX hydrogel to the inflamed irradiated rats showed no significant change in the paw volume. In contrast, the application of TNX-NLCs hydrogel showed potent anti-inflammatory activity and a significant decrease in the percentage of edema by 30%. Similarly, 1 h before carrageenan injection, oral administration of TNX (20 mg/kg) attenuated the paw edema by 25%.

### 3.9. Effect of TNX-NLCs hydrogel against carrageenan-induced hyperalgesia in irradiated rats

The nociceptive threshold means the maximum force applied in grams at a constant rate until the rat withdraws its paw. Three hours after carrageenan injection, the nociceptive threshold decreased significantly by 35% compared to the normal group (*p* < .05). Additionally, the irradiation of rats before carrageenan injection was not significantly different from that observed in the inflamed group (*p* < .05). Furthermore, the application of blank hydrogel and pure TNX hydrogel did not change the nociceptive threshold, compared to the inflamed irradiated rats (*p* < .05). However, either the application of TNX-NLCs or oral administration of TNX showed high analgesic activities with high nociceptive threshold reaching 132–135 g respectively (Figure 6(B)).

### 3.10. Effect of TNX-NLCs hydrogel on histological changes associated with carrageenan-induced paw inflammation in irradiated rats

**Figure 7.**
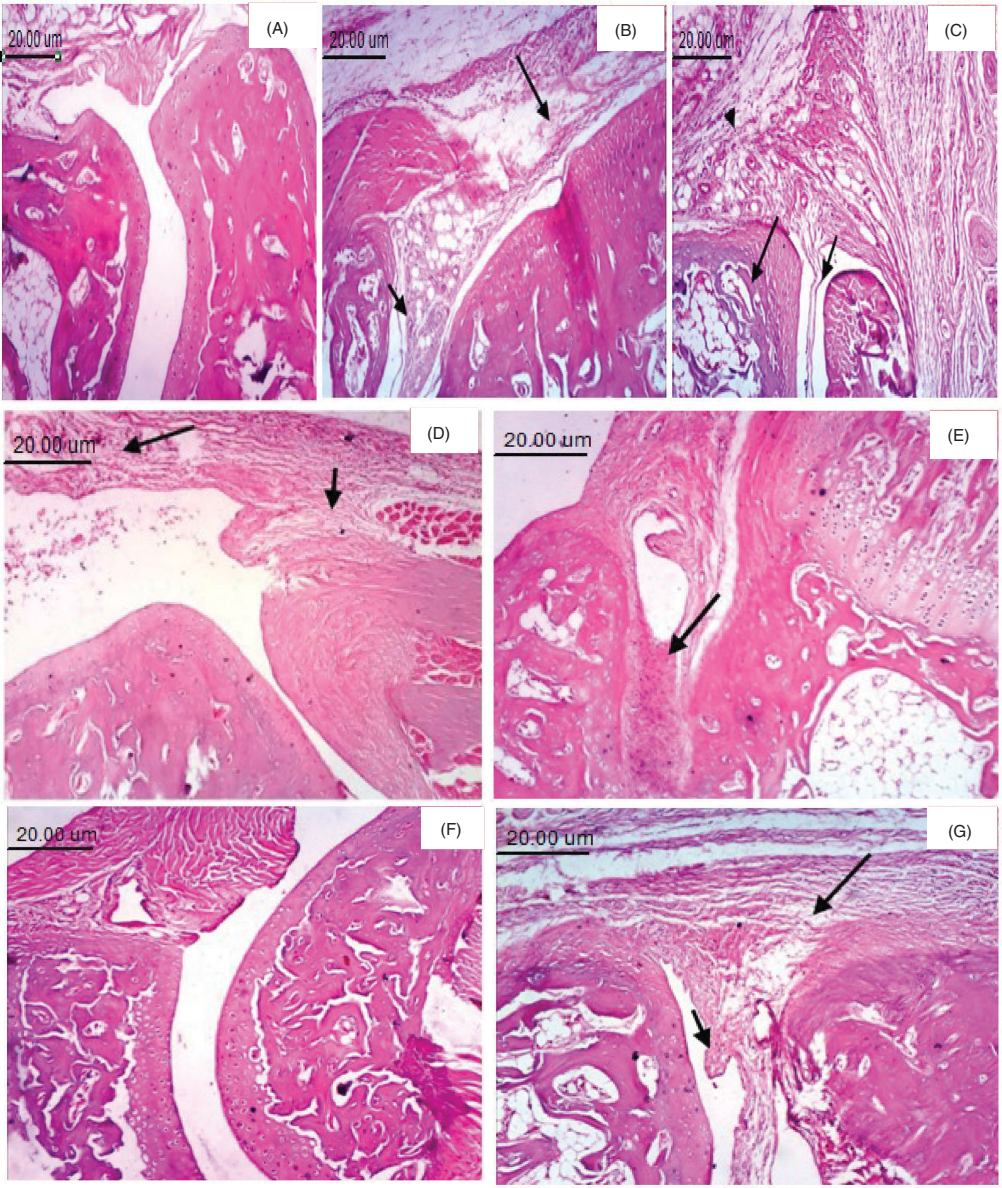
Photomicrographs of paw tissue (H & E, 100×). (A) Normal group, showing two articular cartilages separated by normal joint space with normal synovial membrane. (B) Inflamed group, showing pannus formation (small arrow) and marked edema (large arrow). (C) Inflamed irradiated, showing pannus formation (small arrow), necrosis of cartilage (large arrow) and edema with inflammatory cells infiltration (arrowhead). (D) Inflamed irradiated group treated with blank hydrogel, showing edema (small arrow) and inflammatory cells infiltration (large arrow). (E) Pure tenoxicam (TNX) hydrogel showing the accumulation of inflammatory exudate (arrow). (F) Tenoxicam-nanostructured lipid carriers (TNX-NLCs) hydrogel, showing no histopathological changes. (G) Oral TNX, showing pannus formation (small arrow) and edema (large arrow).

H&E staining examined paw tissue samples from each experimental group to assess histologically the anti-inflammatory effect of TNX-NLCs hydrogel. The normal control group showed two articular cartilages with normal synovial membrane separated by normal joint space (Figure 7(A)). However, significant damage was observed 3 h after carrageenan injection with a noticeable accumulation of infiltrating inflammatory cells and edema (Figure 7(B)). Moreover, radiation exposure before carrageenan induction led to massive destruction in paw tissue (Figure 7(C)). The application of TNX-NLC_S_ hydrogel reduced the morphological alterations to a greater extent than pure TNX hydrogel and oral TNX (Figure 7(D–G), respectively).

### 3.11. Biochemical findings

To evaluate systemic changes in irradiated rats after carrageenan-induced paw inflammation and to what extent TNX-NLCs hydrogel could attenuate that changes, some biochemical parameters relevant to the occurrence of oxidative stress and inflammation were measured.

**Figure 8.**
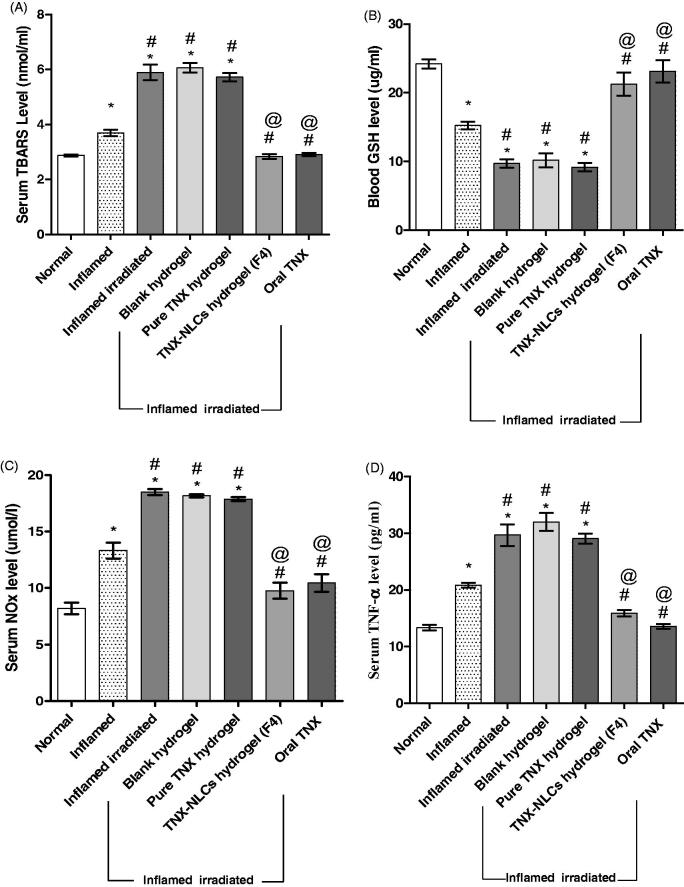
Effect of tenoxicam-nanostructured lipid carriers (TNX-NLCs) hydrogel on (A) the serum level of thiobarbituric acid reactive substances (TBARS, mmol/l), (B) the blood level of reduced glutathione (GSH, mg/ml), (C) total nitrate/nitrite (NOx, µmol/l) and (D) tumor necrosis factor-alpha (TNF-α, pg/ml) in inflamed irradiated rats. Statistical analysis was carried out by one-way ANOVA test. All values were expressed as mean ± s.e.m (*n* = 6). *Denotes statistical significance at *p* < .05 vs normal group. #denotes statistical significance at *p* < .05 vs inflamed group. @denotes statistical significance at *p* < .05 vs inflamed irradiated group.

Injection of carrageenan into the paws of naive rats induced oxidative stress evidenced by a significant increase in TBARS serum level by 27% and a corresponding decrease in the blood level of GSH by 37%. Irradiation of rats before carrageenan induction led to a severe increase in TBARS serum level and a decrease in GSH blood level of about 103 and 60%, respectively. None of these parameters were reversed by blank hydrogel or pure TNX hydrogel. However, both TNX-NLCs hydrogel and oral TNX tended to normalize TBARS and GSH levels (Figure 8(A,B), respectively).

Moreover, the serum level of NOx was increased after carrageenan induction by 62%. The rats exposed to ionizing radiation caused a further increase in the serum level of NOx reaching 125% in comparison to normal. Treatment with either TNX-NLCs hydrogel or oral TNX tended to prevent this increase by nearly 22 and 28%, respectively, compared to normal. Neither blank hydrogel nor pure TNX hydrogel had any influence on the serum level of NOx (Figure 8(C)). Additionally, carrageenan induction in non-irradiated rats increased the serum level TNF-α by 56%. Irradiation of rats by 6 Gy pre-induction of carrageenan resulted in an even higher increase, reaching 123%. Treatment of the inflamed irradiated group by either TNX-NLCs hydrogel or oral TNX markedly guarded against the increase in TNF-α. On the other hand, the application of blank hydrogel and pure TNX hydrogel did not show any significant changes than the inflamed irradiated group (Figure 8(D)).

## 4. Discussion

The transdermal route has been approved for the administration of NSAIDs to avoid the drawbacks of oral administration, such as gastrointestinal symptoms and renal impairment (Cordero et al., 1997). Therefore, the possibility of delivering TNX through the skin for local inflammation is desirable. Many studies have shown that the penetration of TNX through the skin is very poor (Karadzovska et al., 2013; Negi et al., 2014). Hence, pharmaceutical nanotechnology was utilized to enhance therapeutics delivery parameters. The multi-benefits and biocompatible NLCs are a very suitable delivery system for the poorly permeated TNX (Khurana et al., 2015). A secondary vehicle, hydrogel, is utilized to constitute suitable viscosity for a dermal application that aided and extend residence time on the skin. The current study aimed to explore the potential of formulated transdermal nanosized TNX preparation in attenuating the systemic inflammatory reaction in irradiated rats using carrageenan-induced paw edema and hyperalgesia model.

In the present study, high shear homogenization and ultrasonication method are successful in the preparation of TNX-NLCs. The method for preparing NLCs is economical, simple, and reproducible (Fang et al., 2003). Six formulations were prepared and assayed for their particle size, PDI, zeta potential, EE%, and *in-vitro* release. Particle size has a critical influence on potential drug applications. All formulations’ particle sizes indicating a nano-sized scale. However, no relationship was observed between lipid chemical structure and particle size. This could be due to the complex structure of the lipids used (Das et al., 2012). Particle size was increased with an increasing percentage of liquid lipid, formulations F3 and F6 recorded the largest particle size due to the highest liquid lipid content. F3 and F4 exhibited the lowest PDI values but F4 showed the smallest particle size and consequently the largest surface area which might be the main reasons for the highest release rate of TXN from this formulae. High PDI values indicate the heterogeneity of the nanoparticle size while smaller PDI indicates the homogeneity of the nanoparticle size (monodisperse nanoparticles) in suspension. There was no relation between PDI and emulsifiers concentration in all formulations. Zeta potential is considered as the main parameter for the stability of TNX dispersion and its resistance to aggregation. It indicates the electrostatic repulsion degree of the dispersed phase (Algandaby et al., 2016). The liquid lipid present in NLCs affects their EE% to a large extent by creating imperfections in a highly ordered crystal and thus offering sufficient space to effectively load a large amount of drug (Salerno et al., 2010).

To develop a controlled release system with transdermal applicability, it is of great importance to understand the release mechanism and kinetics. In this study, the sample and separate methods were adopted to evaluate the *in-vitro* release of TNX from the investigated formulations. F4 formula exhibited the best release percent in comparison with other formulations of TNX. In addition, the F4 formula was the smallest particle size formula, so it was selected for subsequent experimentation. The kinetics analysis showed that the drug release from nanoparticles follows the diffusion model. Based on this model of drug release, as the soluble amount of TNX increase the release rate increase. Therefore, formatting the drug in the forms of nanoparticles increased the surface area which increased the solubility and the rate of release. It was found that the release mechanism is super case II transport.

Drug release from lipid particles attains by lipid particle degradation inside the body and by diffusion. It might be desirable in some cases to have a controlled fast release going beyond diffusion and degradation. When the particles are administrated this release should be triggered by an impulse. NLCs accommodate the drug due to their highly unordered lipid structures. A burst release can be generated by applying the trigger impulse to the matrix to convert in a more ordered structure. NLCs of certain structures can be triggered this way for example, when applying to the skin the particles incorporated in a hydrogel. An increase in drug release rate due to an increase in temperature and water evaporation and due to very small particle size (Hou et al., 2003).

TEM examination of the F4 formula identified and confirmed TNX-NLCs formation. High shear homogenization and ultrasonication methods can produce TNX NLCs with no phase separation. The FTIR spectrum of F4 formula shows the characteristic peaks of both TNX and emulsifier (Pluronic F68 or F127) which prove the absence of any chemical interaction in the formula.

In order to prolong the persistence of NLCs on the administration site on the skin, it is essential to load the F4 formula in a suitable gelling agent to get the consistency of hydrogel. Furthermore, this reduces the possibility of nanoparticle agglomeration and subsequently increases the particle size dramatically. Else, the release profiles of pure TNX hydrogels were less than that of corresponding hydrogels of TNX-NLCs. It can be concluded that it signifies the importance of optimizing nanoparticulate formulations to improve carrier characteristics. The potential of nanonization in ameliorating dissolution rate and bioavailability is ascribed to a remarkable decrease in particle size with a maximized surface area. Nanonization technology enhances both the dissolution rate and solubility (Hashem et al., 2015). According to the Noyes–Whitney equation, the dissolution rate can be increased with the surface area. Based on earlier research, the Ostwald–Freundlich and Kelvin equations indicate that this no longer applies at the nanoscale particle size, below 1 µm or preferably <0.1 µm, where the extreme curvature of nanoparticles leads to an increase in dissolution pressure and subsequently solubility (Muller & Keck, 2004; Dolenc et al., 2009).

*Ex-vivo* skin permeation study showed that the higher drug permeated could be due to the presence of the lipid material inside the applied hydrogel which can facilitate the drug penetration to stratum corneum. Additionally, the presence of surfactants such as Pluronic F127 and F68 may help in skin deformation and accelerate the drug absorption across the deformed skin. These results strongly confirm the concluded fact that drug nanoparticles loading in gel bases enhanced the skin permeation of the drug (Elmowafy et al., 2017).

To assess the anti-inflammatory and analgesic effects of formula F4 (TNX-NLCs hydrogel), carrageenan-induced paw edema has been used as an acute model for inflammation. The course of acute inflammation induced by carrageenan is biphasic (Vinegar et al., 1969), the initial phase observed within 1 h and starts with the release of histamine, serotonin, and bradykinin from mast cells. While, the delayed phase (after 1 h) is due to the neutrophil infiltration into the inflammatory site and the production of free radicals and pro-inflammatory mediators such as prostaglandins (PGs), NO, and various cytokines such as TNF-α which considered as a major pro-inflammatory cytokine that capable of accelerating the inflammatory process, in addition to IL-1β, and IL-6 (Vinegar et al., 1969; Di Rosa et al., 1971). Accordingly, the measurement of the paw edema, in the present study, was carried out 3 h after the carrageenan inoculation. Since it is the moment when carrageenan maximum effect is manifested and the anti-inflammatory activity of TNX-NLCs hydrogel is best observed.

Several studies have shown that exposure of animals to radiation before induction of inflammation led to an exaggeration of the inflammatory responses and increased release of inflammatory mediators (El-Ghazaly & Khayyal, 1995; Khayyal et al., 2009; El-Ghazaly et al., 2017). As well, the current study demonstrated that irradiation of rats by 6 Gy before inoculation of carrageenan led to a significant increment in the paw volume in comparison to the inflamed non-irradiated group. This effect may be related to the release of inflammatory mediators through the lipoxygenase, cyclooxygenase (COX) pathways, or the release of lysosomal enzymes resulting from disruption of the cell membranes (Trocha & Catravas, 1980; Khayyal et al., 2009). This disruption can be due to direct interaction of cellular membranes with gamma-rays or through an indirect action of ionizing radiation with other atoms or molecules in the cell (particularly water) to produce free radicals that can diffuse deep enough to reach and damage DNA leading to an inflammatory cascade (Reeves, 1999).

The development of inflammation and the generation of pain have a strong connection. Since exposure to radiation caused a significant increase in the volume of the paw, it was therefore expected that the nociceptive threshold would decrease significantly after the exertion of mechanical hyperalgesia on the irradiated paw of rats (Randall & Selitto, 1957; Oka et al., 2007; Zucoloto et al., 2017). Controversially, the present study showed no significant difference between the nociceptive threshold recorded in the inflamed irradiated rats and that observed in the inflamed group. According to Kereškényiová & Šmajda (2004) findings, that effect could be attributed to the release of endogenous opioids after radiation exposure. Similar findings were reported by other authors (El-Ghazaly et al., 2017; Ragab et al., 2017).

Application of pure TNX hydrogel after carrageenan injection did not show any change in the paw volume or nociceptive threshold and that could be attributed to the poor penetration of TNX through the skin (Karadzovska et al., 2013; Negi et al., 2014). On the contrary, TNX-NLCs hydrogel showed a decrease in the paw volume and high analgesic activity. Moreover, histological examination of the paw tissues showed that TNX-NLCs hydrogel reduced the morphological alterations to a greater extent in comparison with pure TNX hydrogel. This effect could be due to particle size reduction, along with increased permeability of TNX-NLCs hydrogel through rat skin as presented in our *ex-vivo* findings.

Seldom of researches has shown carrageenan-induced systemic alterations, for instance, Cicala et al. (2007), Vazquez et al. (2015), and Oka et al. (2007). Besides, Ou et al. (2019) reported an increase in the release of various inflammatory mediators and oxidative stress biomarkers in the blood after the injection of carrageenan.

In conformity to these studies, the present research was directed to investigate the systemic effect of carrageenan in irradiated rats and to what extent the transdermal application of TNX-NLCs hydrogel could attenuate these effects. Our results showed that pure TNX hydrogel did not reverse the increase in TBARS serum levels and the decrease in GSH blood, as well as the increase in NOx and TNF-α serum levels following inoculation of carrageenan in irradiated rats. However, both the hydrogel TNX-NLCs and the oral TNX were guarded against these changes to roughly the same extent. Similar findings were reported by other authors (Goindi et al., 2016; Baranowski et al., 2018; Shah et al., 2019). Although the present study did not measure the pharmacokinetics of the drug, our findings suggested that TNX-NLCs hydrogel reached the dermis of the skin and then passed into the systemic circulation and showed its anti-inflammatory effect almost similar to the oral TNX by decreasing the production of free radicals and pro-inflammatory mediators that were accelerated 3 h after carrageenan inoculation.

## 5. Conclusions

The present study was directed to formulate a hydrogel loaded with nanostructured lipid carriers (NLCs) aiming to improve the transdermal delivery of TNX. Formula F4 was selected from six formulations because it possesses the smallest particle size high, lower PDI, and high release efficiency. TNX-NLCs, which were successfully prepared and characterized for their particle size, PDI, zeta potential, EE, *in-vitro* drug release, and *ex-vivo* skin permeation studies. The results demonstrated that NLCs could serve as safe potential carriers for effective transdermal delivery of TNX. Moreover, the effectiveness of TNX-NLCs was studied *in-vivo* using carrageenan-induced paw edema and hyperalgesia model in irradiated rats. From the *in-vivo* study findings, we could conclude that TNX-NLCs hydrogel had a significant anti-inflammatory and analgesic effect and could be used as an alternative to oral formulation to treat various inflammatory conditions.
